# PCI in Patients With Heart Failure: Current Evidence, Impact of Complete Revascularization, and Contemporary Techniques to Improve Outcomes

**DOI:** 10.1016/j.jscai.2022.100020

**Published:** 2022-04-11

**Authors:** Yousif Ahmad, Mark C. Petrie, E. Marc Jolicoeur, Mahesh V. Madhavan, Eric J. Velazquez, Jeffrey W. Moses, Alexandra J. Lansky, Gregg W. Stone

**Affiliations:** a Section of Cardiovascular Medicine, Yale School of Medicine, New Haven, Connecticut; b University of Glasgow, Glasgow, Scotland; c Centre Hospitalier de l’Universite de Montreal, Montreal, Quebec, Canada; d Division of Cardiology, Columbia University Irving Medical Center/NewYork-Presbyterian Hospital, New York, New York; e Cardiovascular Research Foundation, New York, New York; f St Francis Hospital, Roslyn, New York; g The Zena and Michael A. Wiener Cardiovascular Institute, Icahn School of Medicine at Mount Sinai, New York, New York

**Keywords:** Heart failure, revascularization, percutaneous coronary intervention, coronary artery bypass grafting

## Abstract

Coronary artery disease (CAD) is the most common cause of left ventricular systolic dysfunction (LVSD) and heart failure (HF). Revascularization with coronary artery bypass grafting (CABG) reduces all-cause mortality compared with medical therapy alone for these patients. Despite this, CABG is performed in a minority of patients with HF, partly due to patient unwillingness or inability to undergo major cardiac surgery and partly due to physician reluctance to refer for surgery due to high operative risk. Percutaneous coronary intervention (PCI) is a less-invasive method of revascularization that has the potential to reduce periprocedural complications compared with CABG in patients with HF. Recent advances in PCI technology and technique have made it realistic to achieve more complete revascularization with PCI in high-risk patients with HF, although no randomized controlled clinical trials (RCTs) of PCI in HF compared with either medical therapy or CABG have been performed. In this review, we discuss the currently available evidence for PCI in HF and the association between the extent of revascularization and clinical outcomes in HF. We also review recent advances in PCI technology and techniques with the potential to improve clinical outcomes in HF. Finally, we discuss emerging clinical trial evidence of revascularization in HF and the large, persistent evidence gaps that should be addressed with future clinical trials of revascularization in HF.

## Highlights

• Coronary artery disease is the most common cause of left ventricular systolic dysfunction and heart failure.
• Revascularization with coronary artery bypass grafting reduces all-cause mortality compared with medical therapy alone for these patients, but coronary artery bypass grafting is performed in a minority of patients with heart failure.
• Recent advances in percutaneous coronary intervention technology and technique have made it realistic to achieve extensive revascularization with percutaneous coronary intervention in high-risk patients with heart failure.
• In this review, we discuss currently available trial evidence for percutaneous coronary intervention in heart failure and associations between the extent of revascularization and clinical outcomes. We also review recent advances in percutaneous coronary intervention technology and techniques with the potential to improve clinical outcomes in heart failure. Finally, we discuss emerging clinical trial evidence of revascularization in heart failure and the large, persistent evidence gaps that should be addressed with future clinical trials of revascularization in heart failure.

## Central Illustration

**Figure:**
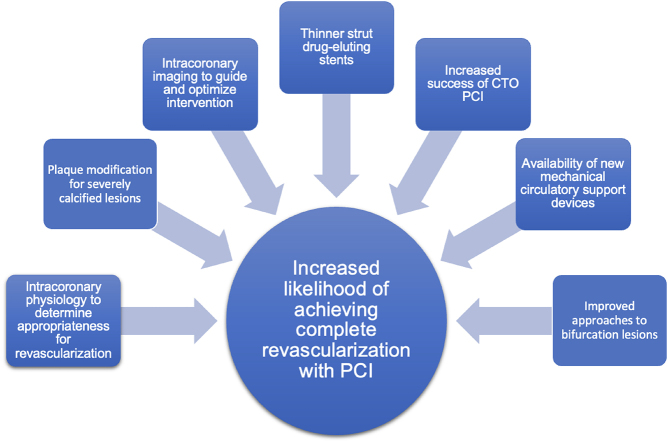

Coronary artery disease (CAD) is the most common cause of heart failure (HF).^1^ The mechanisms by which CAD can cause HF include acute and chronic ischemia and myocardial infarction (MI). Recurrent episodes of ischemia may induce so-called myocardial hibernation, leading to declining left ventricular (LV) function and then consequent clinical HF. The reversal of myocardial hibernation by revascularization is felt to be the key mechanism underpinning potential benefit of revascularization for patients with CAD and HF. A further proposed mechanism of benefit of revascularization in patients with HF is prevention of future myocardial infarction in patients with old infarcts and reduced ventricular function; this may be the mechanism underlying the benefit of coronary artery bypass grafting (CABG) in patients with HF, whereby distal insertion of a bypass graft may provide protection against future proximal vessel stenoses or occlusions.

In the STICH Extension Study, revascularization with CABG in patients with HF with reduced ejection fraction (HFrEF) improved survival compared with medical therapy at a median follow-up duration of 9.8 years.^2^ These benefits did not emerge until longer-term follow-up, and more deaths occurred in the CABG arm than in the medical therapy arm until the 2-year time point.^3^ Despite this mortality benefit, CABG is performed in only a small minority of patients with HF.^4^ This may reflect the high rates of perioperative morbidity and mortality that occur in older patients with HF with multiple comorbid medical problems;^5^ as such, patients and clinicians may be unwilling to accept the up-front hazards associated with CABG despite the longer-term benefits. There are no randomized data for revascularization in patients with HF and preserved ejection fraction (HFpEF).

There is a renewed interest in the potential role of percutaneous coronary intervention (PCI) for patients with CAD and HF. As a less-invasive therapy, PCI might not be associated with the same up-front procedural hazard of CABG in patients with HF and might offer comparable late benefits, especially if extensive revascularization can be achieved. There are currently no randomized controlled trial (RCT) data comparing PCI with either CABG or medical therapy in patients with CAD and HF. In this review, we discuss the currently available evidence for PCI in HF, the impact of complete revascularization (CR) on clinical outcomes in patients with HF, recent advances in PCI technology and techniques that might lead to improved clinical outcomes in HF, the currently emerging clinical trial evidence for revascularization in HF, and the persistent evidence gaps in this area.

## The evidence for PCI in patients with HFrEF

Although there is currently no RCT evidence of the relative merit of PCI compared with either medical therapy or CABG in patients with CAD and HF, other randomized data of PCI for patients with reduced LV ejection fraction (EF) are relevant.

### Evidence for supported PCI in LV systolic dysfunction

#### BCIS-1

The Balloon Pump–Assisted Coronary Intervention Study (BCIS-1) enrolled patients with severe LV systolic dysfunction and CAD.^6^ A total of 301 patients were randomized to elective insertion of an intra-aortic balloon pump (IABP) before PCI or to no IABP insertion. The primary outcome measure was a composite of death, acute myocardial infarction (MI), cerebrovascular event, or further revascularization by PCI or CABG at hospital discharge (capped at 28 days). There was no difference in the primary endpoint between the 2 groups (odds ratio [OR] 0.94, 95% confidence interval [CI] 0.51-1.76, *P* = .85). There was also no difference in all-cause mortality between the 2 groups at 6 months (OR 0.61, 95% CI 0.24-1.62, *P* = .32). However, a mortality benefit favoring the IABP did emerge at a median follow-up of 51 months (hazard ratio [HR] 0.66, 95% CI 0.44-0.98, *P* = .04).^7^ As late mortality was not a pre-specified or powered endpoint, these results should be considered hypothesis-generating.

#### PROTECT II

The randomized PROTECT II trial compared the Impella 2.5 LV assist device (Abiomed Inc) with the IABP in patients undergoing elective high-risk PCI,^8^ including those with unprotected left main disease or last remaining patent coronary artery with an LVEF of ≤35% or 3-vessel disease with an LVEF ≤30%. The primary endpoint was the composite rate of intraprocedure and postprocedure major adverse events at discharge or at 30-day follow-up, whichever was longer. The components of this composite endpoint were all-cause death, nonfatal MI (with procedural MI defined as creatine-kinase-MB or troponin >3× normal), stroke/transient ischemic attack, repeat revascularization, need for cardiac or vascular surgery, acute renal insufficiency, severe intraprocedural hypotension, cardiopulmonary resuscitation, ventricular tachycardia requiring cardioversion or defibrillation, aortic insufficiency, and angiographic failure of PCI. The trial was terminated prematurely for futility after 448 of the 654 expected participants were enrolled. At 30 days, the primary endpoint occurred in 35.1% versus 40.1% of patients assigned to Impella and to IABP, respectively, with no significant difference between the 2 groups (*P* = .28). At 90 days, the composite endpoint of major adverse events occurred in 40.6% versus 49.3% in same groups, again with no significant difference between the group in in the intention-to-treat population (*P* = .066). In the prespecified per-protocol population, defined by patients receiving the assigned support device, the difference in major adverse events at 90 days was statistically significant (51.0% with the IABP versus 40.0% with the Impella, *P* = .02). The difference in all-cause mortality between the 2 groups was not statistically significant at 30 days (5.9% with the IABP versus 7.6% with the Impella, *P* = .47) or 90 days (8.7% with the IABP versus 12.1% with the Impella, *P* = .24). There was no difference in the rate of limb ischemia or the need for cardiac or vascular operations between the 2 groups (*P* = .63), nor was there any occurrence of aortic insufficiency or damage to the aortic valve.

A post hoc analysis of this trial studied reverse LV remodeling in 184 consecutive patients who underwent quantitative echocardiography across both groups.^9^ Reverse LV remodeling was defined as an absolute improvement in the LVEF of ≥5%. Reverse remodeling occurred in 93 of 184 patients (51%), and in this group, the LVEF improved by 13.1% (95% CI 10.2-16.2%, *P* < .001). Reverse remodeling was observed more often in patients treated with extensive 2- or 3-vessel PCI than in those treated with 1-vessel PCI (*P* = .04) or those who underwent only left main PCI (*P* = .28). In a multivariable logistic regression model, patients receiving more extensive revascularization (2- or 3-vessel PCI) were more likely to demonstrate LV remodeling than those receiving limited single-vessel PCI (OR 7.52, 95% CI 1.31-43.25). Patients who demonstrated LV reverse remodeling had significantly fewer events than those without (composite of death, MI, stroke, or transient ischemic attack occurred in 9.7% of patients with reverse LV remodeling compared with 24.2% of those without, *P* = .009). Reverse remodeling was similar in the 2 device groups, and the LVEF improved by 5.9% with the Impella and 6.1% with the IABP (*P* = .92).

Finally, the extent of revascularization was quantified by calculating changes in the myocardial jeopardy score before and after PCI. This analysis demonstrated superior outcomes with extensive compared with limited revascularization, with greater benefit seen among patients treated with the Impella.^10^ These post hoc, nonrandomized data raise the possibility that extensive revascularization (as close to CR as possible) might be an important factor in improving clinical outcomes for patients with CAD and HF.

The results of the PROTECT II trial must be interpreted with caution as the trial was halted prematurely on the advice of the data and safety monitoring board with only 69% of the planned enrollment completed, and the there was no significant benefit detected in the primary endpoint. The BCIS-1 and PROTECT II trials highlight the need for further RCTs to determine if there is a clinical benefit to procedural hemodynamic support for patients with HF undergoing complex PCI.

### Nonrandomized comparisons with CABG

**Figure 1:**
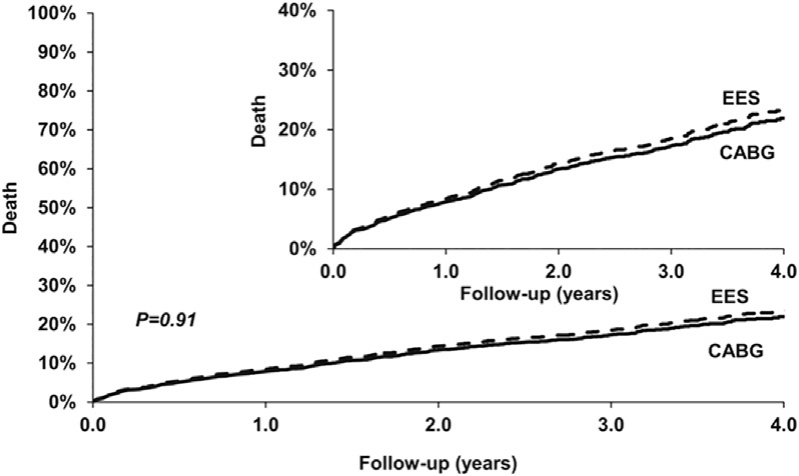
All-cause mortality after treatment of 2126 propensity-matched patients with an LVEF ≤35% with everolimus-eluting stents or CABG from the New York State Registries. CABG, coronary artery bypass grafting; LVEF, left ventricular ejection fraction.

Reproduced with permission from Bangalore et al.^11^

Although there are no RCTs examining the role of PCI in HFrEF, observational studies of the outcomes of PCI vs CABG have been performed. A propensity-matched analysis of 2126 patients from New York State Registries compared outcomes of patients with multivessel disease and an LVEF ≤35% who were treated with either everolimus-eluting stents or CABG.^11^ PCI was associated with a similar risk of death as CABG at 2.9 years (HR 1.01, 95% CI 0.81-1.28; *P* = .91) (Figure 1). PCI was also associated with a lower risk of stroke (HR 0.57; 95% CI, 0.33-0.97; *P* = .04) but a higher risk of both MI (HR 2.16; 95% CI, 1.42-3.28; *P* = .0003) and repeat revascularization (HR 2.54; 95% CI, 1.88-3.44; *P* < .0001). Of note, in patients treated with PCI in whom CR was achieved, there was no longer a significant difference in the risk of MI between the 2 therapies (*P*_interaction_ = .002).

Somewhat different conclusions were reported from a large retrospective observational analysis from Ontario.^12^ This study also included patients with an LVEF ≤35% undergoing either CABG or PCI, with a total of 4794 propensity-matched patients, but the median follow-up was longer at 5.2 years. Patients who underwent PCI had increased rates of all-cause mortality (HR 1.6, 95% CI 1.3-1.7), cardiovascular death (HR 1.4, 95% CI 1.1-1.6), and repeat revascularization (HR 3.7, 95% CI 3.2-4.3). The risk of stroke was lower with PCI than that with CABG (HR 0.7, 95% CI 0.5-0.9).

A recent meta-analysis of 18 studies and 11,686 patients in patients with LV dysfunction^13^ reported similar short-term mortality (30-day mortality) after PCI and CABG (HR 1.18, 95% CI 0.89-1.56, *P* = .25). At longer-term follow-up (12 months or longer), however, there was a lower risk of mortality after CABG than after PCI (HR 0.70, 95% CI 0.43-0.85, *P* < .01). CABG was associated with an increased risk of stroke within 30 days (HR 2.88, 95% CI 1.07-7.77, *P* = .04), but not beyond 12 months (HR 1.18, 95% CI 0.74-1.87, *P* = .49).

The major limitation of these nonrandomized studies is the inability to adjust for unmeasured confounders that might have led to selection of one procedure vs the other. For example, PCI may be most commonly performed for patients with a severely reduced LVEF and multivessel CAD principally when CABG is deemed too high risk or unsuitable for other reasons. This uncertainty highlights the need for randomized data comparing the 2 strategies in patients with a reduced LVEF.

## The evidence for PCI in patients with HFpEF

Epicardial CAD is common in patients with HFpEF. A recent cohort study reported obstructive CAD in half of patients with HFpEF,^14^ consistent with prior autopsy-based studies^15^ and other observational series.^16^ Obstructive epicardial CAD is likely under-recognized in patients with HFpEF, and there are data to suggest that those with obstructive disease have worse outcomes than those without obstructive disease.^14^ There are to date no dedicated randomized trials of revascularization in patients with HFpEF. Whether revascularization with either PCI or CABG can modify the prognosis of patients with HFpEF and obstructive CAD is unknown and warrants assessment in future clinical trials.

## The impact of CR

### Definitions of CR

CR is often the stated goal of coronary revascularization. Various definitions of CR have been used, largely centering on whether all of a patient's “significant” lesions have been treated. The definition of a significant lesion can be based on anatomic, functional, or ischemic parameters, or a combination.^17^ For anatomic criteria, most studies have used visual estimates of diameter stenosis of greater than or equal to 50% or 70% and in vessels with a reference vessel diameter of at least 2.0 mm.^18^ If CR is not achieved, the degree of anatomic incomplete revascularization (ICR) can be quantified by the residual SYNTAX score (rSS) or the residual jeopardy score. These metrics of ICR have been shown to be associated with adverse clinical outcomes.^19^^,^^20^ An alternative approach is to consider functional or ischemic CR, using either invasive physiology or noninvasive tests of ischemia. There have been recent attempts to standardize the definitions of CR, for use both in clinical practice and in clinical research.^21^ This framework includes a gradation, where if the rSS is 0, the patient has anatomic CR, if the rSS is ≥ 9, the patient has anatomic ICR, and if the rSS is between 1 and 8, the patient has “reasonable CR.” Such a framework avoids dichotomizing patients into either CR or ICR as prior studies have shown that reasonable CR is associated with a prognosis intermediate between CR and ICR.^22^, ^23^, ^24^

### CR in patients with HF

There are to date no randomized comparisons examining the impact of CR versus ICR in patients with HF. Achieving CR in patients with HF is often challenging.^25^ This may be due to both anatomic and patient factors. Patients with HF are frequently older with more comorbid conditions than patients without HF and may be unable to tolerate the hemodynamic effects and contrast load of long and complex procedures. Patients with HF also often have CAD that is harder to treat, with increased frequency of chronic total occlusions (CTOs), multivessel disease, and diffuse disease.^26^^,^^27^ There are observational data suggesting that patients with HF with a history of PCI who have ICR have greater all-cause mortality than those who have CR,^28^ with residual coronary stenoses being associated with an increased risk of all-cause mortality.

Achieving CR may be an appropriate target to improve clinical outcomes in patients with HF, but this hypothesis requires investigation in an appropriately designed RCT.

### Outcomes based on CR in patients without HF

The benefit of CABG compared with PCI in complex multivessel disease is often attributed to the greater likelihood of CABG achieving CR. In the SYNTAX trial, CR was achieved more frequently with CABG than with PCI, but long-term mortality was similar after both PCI and CABG in patients in whom CR was achieved.^29^ However, only 2% of the SYNTAX trial population included patients with HF, so these results cannot necessarily be generalized to patients with HF. It should also be noted that these trials were not true randomized comparisons of CR versus ICR.

True randomized comparisons of CR versus ICR exist in the context of ST-segment elevation MI and multivessel disease, with improved outcomes with CR,^30^ but these data cannot be indiscriminately applied to patients with HF undergoing elective revascularization. In the COMPLETE trial^31^ (the largest trial to date examining the impact of CR in patients with ST-elevation myocardial infarction and multivessel PCI), a significant reduction in the primary endpoint of cardiac death or MI (HR 0.49, 95% CI 0.32-0.74) was observed in the subgroup of 794 patients with an LVEF <45%, with no significant interaction between reduced vs preserved LVEF and the incidence of the primary endpoint (*P*_interaction_ = .13). However, subgroup analyses are underpowered; the results should thus be considered hypothesis-generating only and are not evidence for improved outcomes of CR in patients with HF.

## The role of viability and ischemia

The clinical utility of ischemia testing and viability testing to guide revascularization in HF has not been established in RCTs. In the viability substudy of STICH, the presence or absence of viability did not identify patients with differential treatment benefits from revascularization with CABG.^32^^,^^33^ Patients with myocardial viability (which was dichotomized as present or absent in STICH) had improvements in ventricular function, but this was achieved irrespective of treatment with CABG and was not associated with mortality. Caveats do exist with regard to the viability substudy of STICH. First, viability testing was not performed on a randomized subgroup but was performed as per the clinical judgment of the participating investigator, which necessarily introduces bias. Second, viability was classified as either present or absent. Third, only single photon emission computed tomography or dobutamine echocardiography was used; no data therefore exist for positron emission tomography or cardiac magnetic resonance assessment of viability. The role of these newer modalities will at least partially be evaluated in the AIMI-HF study, where patients will be randomized to either single photon emission computed tomography or “advanced” imaging modalities, which are cardiac magnetic resonance or positron emission tomography.^34^ It should be acknowledged, however, that subsequent revascularization is not randomized on the basis of the imaging findings but is at the discretion of the treating clinical team.

In clinical practice, viability testing might not necessarily be used as a binary decision-making aid to support or refute revascularization, but might be used to help guide targeted revascularization (avoiding revascularization in nonviable territories).

Similarly to viability, in a substudy of the STICH trial the presence or absence of inducible ischemia did not identify patients with worse prognoses or select patients for greater beneficial effect of revascularization with CABG.^35^ The principal findings of the ISCHEMIA (International Study of Comparative Health Effectiveness With Medical and Invasive Approaches) trial do not apply to patients with severely reduced ventricular function because they were excluded from the trial, although a substudy of patients with an EF ≤45% and history of symptomatic HF did suggest better outcomes with invasive over conservative therapy.^36^ These findings must be considered hypothesis-generating only in view of the nonrandomized nature and small number of patients.

Until more definitive randomized data are available, the degree of ischemia and viability could be incorporated into clinical decision-making for patients with CAD and HF, but should not be the sole determinant in select patients for revascularization or deferring revascularization.

## Is now the time for RCTs of PCI in patients with ischemic HF?

There have been many important advances in the field of coronary intervention over the past decade, but these have largely been assessed in populations that have excluded patients with HF. These advances include the use of more potent antiplatelet therapy (although this may be associated with greater risks to patients with HF who are often at high bleeding risk and have baseline anemia), intracoronary physiology to guide lesion selection in PCI (although this approach has not yet been validated in patients with HF), ultrathin strut stents, the use of intravascular imaging to optimize stent outcomes, the use of new techniques to recanalize CTOs, optimal approaches to calcified lesions and bifurcations, and the use of percutaneous mechanical circulatory support (MCS) devices to enable high-risk PCI. Several of these bear more discussion as they relate to PCI in patients with HF.

It is also important to emphasize the role of guideline-directed medical therapy at this point. Any potential trial of coronary intervention for HF should be conducted on the background of optimal medical therapy, in a manner similar to the COAPT trial for transcatheter edge-to-edge mitral valve repair for functional mitral regurgitation.^37^ The COAPT trial should be considered emblematic for how a trial of an interventional approach appreciated the synergistic relationship between any potential intervention and background optimized guideline-directed medical therapy.

### Physiology-guided PCI

The role of physiology has not yet been established for patients with HF, and increased filling pressures may affect the reliability of hyperemic pressure indices. The original calculations for fractional flow reserve (FFR) included right atrial pressure, but over the years this has been removed for the sake of simplicity.^38^ The FAME-2 trial initially demonstrated superiority of FFR-guided PCI compared with medical therapy for patients with hemodynamically significant lesions for the primary composite outcome of death, MI, or urgent revascularization.^39^ These initial results, at a mean follow-up of 7 months, were driven by reduced rates of unplanned revascularization, but long-term follow-up at 5 years suggested a reduction in MI (HR 0.66, 95% CI 0.43-1.00).^40^ This trial excluded patients with an LVEF <30%. The superiority of physiologically guided PCI compared with angiographically guided PCI had already been established by the FAME trial,^41^ although <10% of patients had a reduced LVEF. The recent FAME 3 trial results reported that FFR-guided PCI was not noninferior to CABG for patients with 3-vessel CAD for the 1-year endpoint of death, MI, or stroke.^42^ However, only 18% of patients in FAME 3 had an LVEF ≤50%, and patients with an LVEF <30% were excluded, so this trial again has limited applicability to patients with HF (although the *P*_interaction_ was nonsignificant for the subgroup of patients with a reduced versus normal LVEF).

The emergence of nonhyperemic indices of coronary stenosis severity is hoped to increase adoption of intracoronary physiology by removing barriers to implementation by obviating the need for pharmacological vasodilatory agents. The instantaneous wave-free ratio (iFR) has been shown to provide equivalent outcomes to FFR in 2 large-scale RCTs totaling 4529 patients.^43^^,^^44^ These trials did not exclude patients with HF, but only 6% had a history of congestive HF, and 19% had a reduced LVEF. Resting indices have a theoretical advantage over hyperemic indices for patients with HF, as they are not as impacted by elevated filling pressures. Whether these theoretical benefits impact clinical outcomes in patients with HF is unknown. Finally, noninvasive physiologic lesion assessment, including FFR-CT^45^^,^^46^ and QFR (angiography-based FFR),^47^ may obviate the need for invasive physiologic lesion assessment entirely.

### Intracoronary imaging to optimize PCI results

Stent optimization by intracoronary imaging with either intravascular ultrasound (IVUS) or optical coherence tomography (OCT) has been demonstrated to improve outcomes in numerous RCTs but has not been formally evaluated in patients with HF. A meta-analysis of 8 RCTs and 3276 patients with complex coronary lesions compared outcomes with IVUS-guided PCI versus angiography-guided PCI.^48^ IVUS-guided PCI was associated with substantial reductions in major adverse cardiac events (risk ratio [RR] 0.64, 95% CI 0.51-0.80, *P* = .0001), target lesion revascularization (RR 0.62, 95% CI 0.45-0.86, *P* = .004), and target vessel revascularization (RR 0.60, 95% CI 0.42-0.87, *P* = .007). A larger analysis of 10 RCTs (N = 5007 patients) comparing drug-eluting stent (DES) implantation with IVUS versus angiography guidance also demonstrated a 49% reduction in cardiovascular mortality with IVUS (RR 0.51, 95% CI 0.27-0.96, *P* = .04).^49^ Analyses that also included observational studies have demonstrated significant reductions in all-cause mortality.^50^^,^^51^ The role of OCT will be determined in the ongoing ILUMIEN IV trial (NCT03507777), but pooled analyses of RCTs and adjusted observational studies suggest equivalent outcomes to IVUS.^51^^,^^52^

### Newer stent designs

Second-generation DES platforms provide excellent clinical outcomes with superior safety and efficacy to the first-generation DES. They have been considered the standard of care, and their results have not been improved on by various technological iterations including bioresorbable scaffolds, polymer-free DES, and bioresorbable polymer-based DES. Recently, ultrathin strut DES have demonstrated incremental improvements in clinical outcomes over second-generation DES. In a meta-analysis of 16 trials and 20,701 patients,^53^ with a weighted mean follow-up of 2.5 years, ultrathin-strut DES were associated with a 15% reduction in target lesion failure and a 25% reduction in target vessel failure; these were driven by reductions in target lesion and target vessel revascularization. There were also numerically fewer instances of MI and stent thrombosis.

Thinner strut stents and the use of intracoronary imaging to optimize PCI results may also permit shortening the duration of dual antiplatelet therapy, which is particularly relevant for patients with HF who are often at high bleeding risk.

### Improvements in CTO recanalization

There are no randomized data pertaining to CTO PCI in patients with HF. The lower rate of CR with PCI than with CABG in patients with multivessel disease is most commonly due to the presence of a CTO. CTO PCI is seldom attempted, and the success rate of CTO PCI in the SYNTAX trial was approximately 50%.^54^ In the EURO-CTO RCT, CTO PCI led to greater symptomatic improvement and quality of life than medical therapy.^55^ However, the mean LVEF in this trial was 55%, so these results do not apply to patients with HF. Moreover, no CTO RCT has demonstrated reduced mortality, although all trials to date have been underpowered in this regard. The only data demonstrating an improved LVEF after CTO PCI in patients with HF are observational in nature and, therefore, subject to bias.^56^

CTO PCI is associated with inherent procedural risk, and procedural complications may be amplified in patients with HF. There has been significant recent advancement in both technique and technology for CTO PCI. The procedure has become increasingly protocolized, with widespread adoption of the “hybrid” algorithm,^40^ which provides a framework for how cases can be approached. Concurrent with the development of these algorithms, there have been improvements in devices, including guidewires and microcatheters. The combination of these factors has allowed expert operators to achieve 85% to 90% success rates.^57^^,^^58^ These success rates must be balanced against potentially increased procedural risk in certain patient and lesion subsets, particularly when the retrograde approach is used.^59^ The impact of contemporary CTO PCI in patients with HF will need to be evaluated in appropriately designed prospective RCTs.

### MCS

The availability of advanced percutaneous MCS devices, such as Impella (Abiomed), TandemHeart (LivaNova, Inc), and veno-arterial extracorporeal membrane oxygenation, can facilitate PCI in patients with reduced LV systolic function. Their use in this setting can enable more extensive and complete revascularization.^10^ There is currently no RCT evidence clearly demonstrating the safety and efficacy of these devices for patients with HF. The data for Impella from the PROTECT II RCT was discussed previously, and future trials of MCS devices are described in the following sections. The only RCT data for TandemHeart are from 2 small trials in cardiogenic shock.^60^^,^^61^ Veno-arterial extracorporeal membrane oxygenation has also not yet been evaluated in an RCT of patients with HF; the only study evidence is for patients with acute MI and cardiogenic shock^62^ and for those with out-of-hospital cardiac arrest and refractory ventricular fibrillation.^63^

### The cumulative impact of multiple advances in PCI

Some of these advances have been demonstrated to individually improve outcomes when assessed in patients without HF (see Central Illustration). Their clinical utility in patients with HF requires further study, and it may be that they are best appraised collectively as part of a “contemporary PCI” strategy rather than in isolation.

The SYNTAX II study^64^ was a multicenter, single-arm study that included patients with 3-vessel disease. The aim of the study was to explore whether the integration of multiple new developments in PCI practice might improve clinical outcomes compared with those achieved in the original SYNTAX trial. These contemporary PCI practices included intracoronary physiology with a hybrid iFR/FFR approach to define appropriateness of revascularization of all target lesions, implantation of a thin-strut platinum–chromium DES, mandated post-PCI IVUS to optimize stent deployment in accordance with predefined criteria for expansion and apposition, treatment of bifurcation lesions in accordance with the European Bifurcation Club consensus^65^, and revascularization of CTOs using contemporary techniques by a dedicated CTO operator.^57^

The 5-year results of the SYNTAX II study have recently been published.^66^ The success rate of CTO PCI in this study was 87%, permitting more CR than that achieved in the SYNTAX I PCI cohort. The rate of major adverse cardiac and cerebrovascular events at 5 years was lower in the SYNTAX II cohort than that in the matched SYNTAX trial PCI cohort (21.5% versus 36.4%; HR 0.54, 95% CI 0.41-0.71, *P* < .001). The difference in all-cause mortality was now significant at the 5-year time point (8.1% versus 13.8%; HR 0.57, 95% CI 0.37-0.90, *P* = .013), driven by differences in cardiac death (2.8% versus 8.4%; HR 0.32, 95% CI 0.16-0.64, *P* < .001). When compared with the CABG cohort from the SYNTAX trial, there were no significant differences in the rates of major adverse cardiac and cerebrovascular events (21.5% versus 24.6%; HR 0.87, 95% CI 0.64-1.17, *P* = .35) or all-cause mortality at 5 years (8.1% versus 10.8%; HR 0.74, 95% CI 0.46-1.19; *P* = .21). A summary of the event rates at 5 years in the SYNTAX II study along with the matched PCI and CABG cohorts of the SYNTAX trial is shown in Table 1.

**Table 1:** Summary of clinical event rates in the SYNTAX II study and the equipoise-derived PCI cohort and CABG cohort of the SYNTAX trial.

Clinical event	SYNTAX II study (N = 454)	SYNTAX trial		SYNTAX II vs SYNTAX PCI cohort		SYNTAX II vs SYNTAX CABG cohort	
		PCI cohort (N = 315)	CABG cohort (N = 334)	HR (95% CI)	*P* value	HR (95% CI)	*P* value
MACCE	21.5% (96/454)	36.4% (112/315)	24.6% (76/334)	0.54 (0.41-0.71)	<.001	0.87 (0.64-1.17)	.35
All-cause mortality	8.1% (36/454)	13.8% (42/315)	10.8% (33/334)	0.57 (0.37-0.90)	.013	0.74 (0.46-1.19)	.21
Stroke	2.3% (10/454)	2.7% (8/315)	3.3% (10/334)	0.83 (0.33-2.12)	.70	0.68 (0.28-1.63)	.39
Myocardial infarction	2.7% (12/454)	10.4% (31/315)	2.5% (8/334)	0.26 (0.13-0.50)	<.001	1.04 (0.43-2.55)	.93
Repeat revascularization	13.8% (60/454)	23.8% (70/315)	12.6% (37/334)	0.56 (0.39-0.78)	<.001	1.14 (0.76-1.72)	.53

CABG, coronary artery bypass grafting; CI, confidence interval; HR, hazard ratio; MACCE, major adverse cardiac and cerebrovascular events; PCI, percutaneous coronary intervention.

It should be acknowledged that SYNTAX II was a single-arm nonrandomized study, and comparisons with both the matched PCI and CABG cohort from the SYNTAX trial should be considered exploratory. Furthermore, patients included did not have LV systolic dysfunction; the mean LVEF in SYNTAX II was 58.1 ± 8.3%, and in the SYNTAX trial comparator arm, it was 61.8 ± 11.3%. Whether these results apply to patients with a reduced LVEF is therefore unknown. Although the absence of a randomized control group precludes any definitive conclusions on effect of therapy, the favorable results of SYNTAX II reinforce the need for an RCT incorporating contemporary PCI techniques in a mandated, protocolized fashion compared with either standard of care PCI or CABG.

## Renewed interest in the field of complex and high-risk PCI

**Figure 2:**
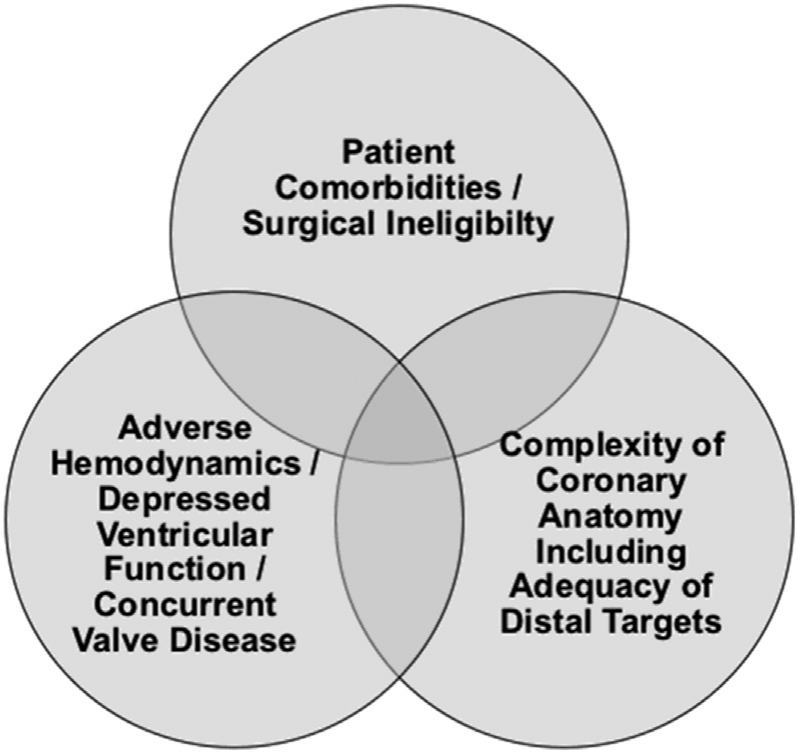
Patient risk for percutaneous coronary intervention is determined by these 3 potentially overlapping and additive domains.

Adapted with permission from Kirtane et al.^67^

Whether the results of the SYNTAX II study would apply to a population of patients with HF is currently unknown; however, there has been recent interest within the interventional community of offering indicated percutaneous revascularization to the highest-risk subset of patients who often have severe LV systolic dysfunction (Figure 2). These have frequently been referred to as “complex higher-risk and indicated procedures” (CHIP).^67^ Alongside advances in device technology, a new focus on extended dedicated training and education on advanced interventional techniques have led to improvements in procedural success by highly skilled operators treating the most complex lesion subsets and highest-risk patients (those with HF). Operator experience is a well-established factor affecting outcomes for CTO PCI,^68^^,^^69^ left main PCI,^70^^,^^71^ and atherectomy,^72^ and operator volume and experience appear to be most strongly related to outcomes in complex PCI rather than more straightforward lesions.^73^^,^^74^

**Central Illustration:**
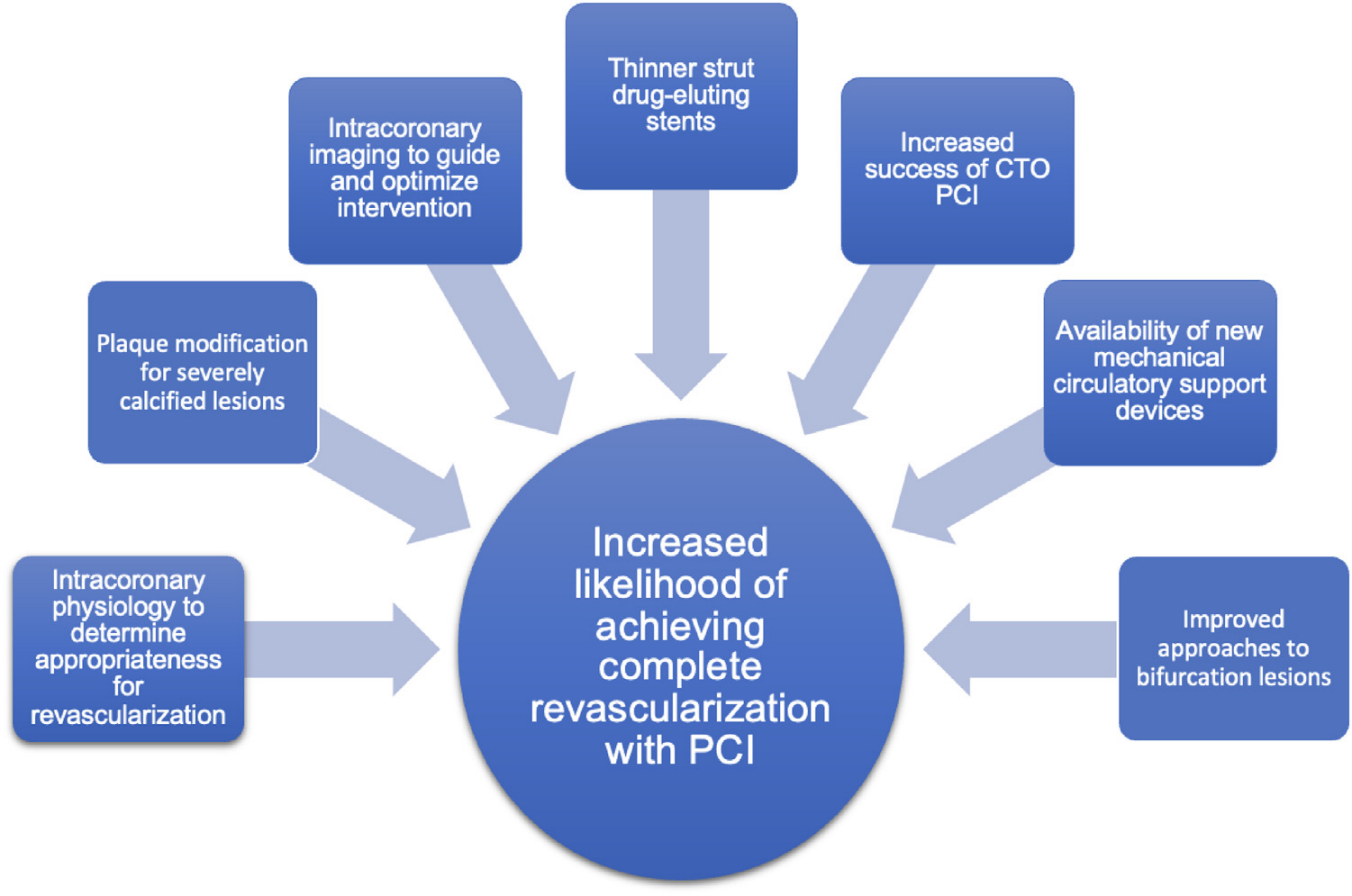
Summary of Recent Advances in Both Technology and Technique for Percutaneous Coronary Intervention. With these advances, it is now realistic to safely achieve complete revascularization with PCI in many complex lesion subsets and high-risk patients with heart failure. These approaches require dedicated evaluation in patients with heart failure to establish whether they lead to improved outcomes. CTO, chronic total occlusion; PCI, percutaneous coronary intervention.

A key determinant of achieving optimal outcomes is appropriate patient and lesion selection. Any potentially deleterious effects of PCI (flow-limiting dissections, side-branch occlusion, bleeding and vascular complications) will be magnified and potentially of greater adverse impact in patients with HF. Despite the fact PCI is a less-invasive therapy than CABG, there still exists a very real potential for harm if patient selection or case planning is suboptimal.

The synergy between improved technologies and technique may allow complex patients with HF to be revascularized with PCI with high rates of success and reduced complications, achieving durable long-term benefits. Such an approach might be able to mitigate the increased early mortality hazard observed with CABG while maintaining its observed long-term mortality benefits.^3^^,^^75^ The relative performance of contemporary PCI compared with both CABG and medical therapy needs to be evaluated in appropriately powered RCTs conducted at expert CHIP centers.

## Emerging evidence and evidence gaps

Several ongoing RCTs are evaluating PCI in patients with HF (Table 2). The REVIVED-BCIS2 trial in the United Kingdom has randomized 700 patients with severe CAD, LVEF ≤35%, and myocardial viability to either PCI with CR encouraged vs medical therapy alone.^76^ The primary endpoint is a composite of all-cause mortality or HF hospitalization, and results are expected in 2022.

**Table 2:** Ongoing or planned randomized trials evaluating percutaneous coronary intervention in patients with heart failure.

Study name	Inclusion criteria	Treatment arm	Control arm	Projected sample size	Primary endpoint	Planned follow-up
CHIP-BCIS3	Patients with extensive coronary disease and an LVEF ≤35% scheduled to undergo complex PCI	PCI with LV unloading; the pLVAD inserted at the start of the procedure	PCI without pLVAD. Alternative mechanical circulatory support devices such as IABP or ECMO will only be permitted in case of complications	250	Composite hierarchical outcome including death, stroke, spontaneous MI, cardiovascular hospitalization, or periprocedural MI analyzed using a win ratio method	Minimum 12 months (up to 4 years)
PROTECT IV	Patients with a chronic coronary syndrome or NSTEMI with an LVEF ≤40% or STEMI ≥24 hours and <30 days after the symptom onset with an LVEF ≤30%, planned for complex PCI after heart team discussion	Impella-supported PCI	Standard-of-care PCI with or without the IABP	1252	Composite of all-cause death, stroke, MI, or hospitalization for cardiovascular causes	All patients will have follow-up for 3 years after randomization. The primary endpoint will be assessed after the last randomized patient reaches 1-year follow-up.
REVIVED-BCIS2	All of the following: LVEF ≤35%, extensive coronary disease, viability in at least 4 dysfunctional segments that can be revascularized by PCI	PCI and medical therapy	Medical therapy alone	700	Composite of all-cause death or hospitalization for heart failure	Patients will be followed for at least 2 years from randomization (expected range: 2-8.5 y).

ECMO, extracorporeal membrane oxygenation; IABP, intra-aortic balloon pump; LV, left ventricular; LVEF, left ventricular ejection fraction; MI, myocardial infarction; PCI, percutaneous coronary intervention; pLVAD, percutaneous LV assist device; STEMI, ST-elevation myocardial infarction.

The ongoing PROTECT IV trial (NCT04763200) is randomizing 1252 patients with complex CAD and LV systolic dysfunction to Impella-supported PCI vs standard of care PCI (IABP or no MCS). Participants are those with chronic coronary syndromes or non–ST-segment elevation acute coronary syndromes with an LVEF ≤40% and those with ST-segment elevation MI and an LVEF ≤30%. All patients in the trial will undergo PCI, with a goal of achieving CR. The primary endpoint is a composite of all-cause mortality, stroke, durable LVAD or heart transplant, MI, or hospitalization for cardiovascular causes at 3-year follow-up.

CHIP-BCIS3 (NCT05003817) in the United Kingdom is randomizing 250 participants with an LVEF ≤35% and extensive CAD who are undergoing complex PCI to either LV unloading during PCI with a percutaneous LV assist device (pLVAD) or control (high-risk PCI without pLVAD). The choice of the device will be at the discretion of the operator. The use of an IABP is not classified as pLVAD in the context of this trial. The primary endpoint in this trial is a composite hierarchical outcome of death, stroke, spontaneous MI, cardiovascular hospitalization, or periprocedural MI at a minimum 12-month follow-up.

Despite these ongoing trials, there are persistent evidence gaps in patients with LV systolic dysfunction. The PCI versus medical therapy in HFrEF evidence gap will be partially answered by the REVIVED-BCIS2 trial. To our knowledge, there is no current or planned randomized comparison of PCI versus CABG for patients with HFrEF. Moreover, there are no randomized data whatsoever for revascularization (whether by PCI or CABG) vs medical therapy in patients with HFpEF. RCT evidence is also needed to establish the benefit of CR with contemporary PCI techniques (similar to those used in SYNTAX II) compared with standard of care PCI or culprit-only PCI for patients with HF. There are also important evidence gaps with regard to the impact of revascularization on HF hospitalization and symptomatic status. Hospitalizations and quality-of-life metrics are important to patients with HF, and we lack randomized data in this domain. The upcoming REVIVED-BCIS2 trial will examine the impact of PCI on HF hospitalization and will also report data with regard to quality-of-life scores such as the Kansas City Cardiomyopathy Questionnaire and EuroQol EQ-5D-5 L.

Such trials should provide data with regard to survival between the assessed competing therapies, but also other important outcomes with regard to quality-of-life and HF hospitalization as well as stroke and neurocognitive endpoints which might be expected to substantially differ between PCI, CABG, and medical therapy.

## Conclusions

Despite the proven long-term survival benefit of CABG for patients with CAD and HFrEF, fewer than 10% of such patients undergo CABG owing to prohibitively high surgical risk or an unwillingness to accept the early procedural hazards of surgery. Revascularization with contemporary PCI techniques aiming for CR in patients with HFrEF may provide similar long-term mortality as CABG without the early hazards, expanding revascularization access for patients with HF and CAD. To date, however, comparative data to establish the role of PCI compared with CABG in patients with HF are lacking. The time has come to formally appraise the role of lesser-invasive contemporary PCI compared with CABG in patients with HF and close the remaining evidence gaps.

### Declaration of competing interest

The authors declared no potential conflicts of interest with respect to the research, authorship, and/or publication of this article.

## Ethics statement

This research has adhered to the relevant ethical guidelines.

## Peer review statement

Given her role as Editor in Chief, Alexandra Lansky had no involvement in the peer review of this article and has no access to information regarding its peer review. Full responsibility for the editorial process for this article was delegated to Dean J. Kereiakes.
